# Macrophage Autophagy in Atherosclerosis

**DOI:** 10.1155/2013/584715

**Published:** 2013-01-21

**Authors:** Maria Chiara Maiuri, Gianluca Grassia, Andrew M. Platt, Rosa Carnuccio, Armando Ialenti, Pasquale Maffia

**Affiliations:** ^1^Department of Experimental Pharmacology, University of Naples Federico II, 80131 Naples, Italy; ^2^INSERM U848, Institut Gustave Roussy, 94805 Villejuif, France; ^3^Institute of Infection, Immunity and Inflammation, College of Medical, Veterinary and Life Sciences, University of Glasgow, 120 University Place, Glasgow G12 8TA, UK

## Abstract

Macrophages play crucial roles in atherosclerotic immune responses. Recent investigation into macrophage autophagy (AP) in atherosclerosis has demonstrated a novel pathway through which these cells contribute to vascular inflammation. AP is a cellular catabolic process involving the delivery of cytoplasmic contents to the lysosomal machinery for ultimate degradation and recycling. Basal levels of macrophage AP play an essential role in atheroprotection during early atherosclerosis. However, AP becomes dysfunctional in the more advanced stages of the pathology and its deficiency promotes vascular inflammation, oxidative stress, and plaque necrosis. In this paper, we will discuss the role of macrophages and AP in atherosclerosis and the emerging evidence demonstrating the contribution of macrophage AP to vascular pathology. Finally, we will discuss how AP could be targeted for therapeutic utility.

## 1. Introduction

Atherosclerosis-related cardiovascular diseases are the leading cause of mortality worldwide. In addition to lipid dysfunction and arterial lipid accumulation, immune-inflammatory responses are major factors in directing the initiation and development of atherosclerosis [1, 2]. Macrophages play a central role in each stage of disease pathogenesis [3]. Interestingly, recent investigation into macrophage autophagy (AP) has demonstrated a novel pathway through which these cells contribute to vascular disease [4–7]. In this paper, we will discuss the role of macrophages and AP in atherosclerosis and the contribution of macrophage AP to vascular pathology. Finally, we will discuss how AP could be targeted for therapeutic utility in atherosclerosis.

## 2. The Origin of Vascular Macrophages

Macrophages are defined as diverse, scavenging, and bactericidal tissue-resident cells with critical immune functions. They are present in every endothelial and epithelial surface of the body, exhibit stellate morphology, and express markers including F4/80, CD11b, CD115, macrosialin (CD68), and CD83. They also express an array of Fc receptors, receptors for complement components, scavenging receptors, and pathogen recognition receptors such as Toll-like receptors (TLRs) and Nod-like receptors (NLRs). When activated, tissue macrophages phagocytose and kill microorganisms and secrete proinflammatory cytokines. In addition, the proinflammatory cytokines and chemokines they release upon activation contribute to the recruitment and activation of lymphocytes. However, it is these very functions that drive their well-established role in inflammatory conditions such as atherosclerosis.

The origin of tissue macrophages has been receiving much attention recently, with many long-held concepts proving incorrect. Indeed, many tissue macrophage populations do not arise from blood monocytes but maintain themselves locally in tissues after they are seeded by yolk sac macrophages [8, 9]. However, to our knowledge, the origin of vascular macrophages in the steady state is unclear and during inflammation, it is clear that input from circulating monocytes is critical [10]. Monocytes originate from common CSF-1R^+^CX3CR1^+^Flt3^+^ macrophage/dendritic cell precursors (MDPs) [11] and expand in response to macrophage colony-stimulating factor (M-CSF) [12]. Monocytes in the mouse can be divided into 2 subsets, classical (Ly6Chi CCR2+) and nonclassical monocytes (Ly6Clo CCR2lo) [13], with analogous subsets present in humans [14]. Classical monocytes exit the bone marrow in a CCR2-dependent manner to seed sites of inflammation [15], whereas it is as yet unclear how and if nonclassical monocytes arise from the bone marrow [16].

A central feature of atherosclerosis is the accumulation in the lesion of monocyte-derived, lipid-laden macrophages termed foam cells and, indeed, monocyte recruitment into plaques is critical for, and increases with, disease progression [10, 17, 18]. Consistent with this, mice deficient in M-CSF-derived macrophages (op/op) have reduced development of atherosclerosis [19]. However, perhaps the most compelling evidence of the role of monocyte-derived cells in atherosclerosis is borne out of successful therapeutic studies in mice targeting chemokine/chemokine receptors critical for monocyte chemoattraction to the plaque [20, 21]. Activation of blood vessel endothelium results in the arrest and extravasation of circulating monocytes into the plaque [22], and the extent of recruitment is regulated at least in part by blood monocyte levels [23]. Hypercholesterolemia correlates with an increase in the frequency of classical monocytes, and it is primarily this subset of monocytes that seeds the plaque [24]. Nevertheless, the capacity of nonclassical monocytes to patrol blood vessel walls [11] could be pertinent to the inflammatory process during atherosclerosis, and indeed this subset has been demonstrated to enter plaques [25, 26].

## 3. The Role of Macrophages in Atherosclerosis

Upon entry into the vascular wall, monocytes undergo maturation into macrophages that are critical for the inflammatory response. Although there is undoubtedly heterogeneity in plaque macrophages, the majority of macrophages in the plaque are classically rather than alternatively activated, and this is discussed in recent reviews [27]. Concomitant with this maturation process, macrophages engulf vast amounts of lipid in the form of apoB-containing lipoproteins into membrane-bound droplets to form foam cells [3]. Macrophages utilize scavenger receptors like CD36 and scavenger receptor type A *inter alia* to recognize modified low-density lipoproteins (LDL) [28, 29], and uptake of oxLDL alone can drive inflammatory gene expression in macrophages through a novel recognition pathway involving a CD36-dependent TLR4-TLR6 heterodimer [30]. In addition, signals through scavenging and c-type lectin-like receptors, TLRs, and numerous intracellular sensors can drive macrophage activation. Intriguingly, several endogenous ligands for TLRs such as heat shock proteins (HSPs) can be found in high concentration in atherosclerotic plaques [31] and have been proposed as a potential pathway to activate macrophages and perpetuate atherosclerosis [32]. Interestingly, recent work focusing on the effects of cholesterol crystals on macrophages has uncovered a novel pathway of macrophage activation in atherosclerosis [33, 34]. The intracellular apparatus consisting of NLRP3, ASC, and caspase-1 all cooperate to drive the generation and subsequent release of active IL-1*β* and IL-18 by macrophages in response to cholesterol crystals and play an important role in the development of atherosclerosis [33]. Thus, there are a plethora of described pathways, and additional putative mechanisms, that drive macrophage activation during atherosclerosis.

Once activated, macrophages produce an array of proinflammatory cytokines such as TNF*α*, IL-12, IL-6, IL-1*β* [35], and leukotrienes [36] that drive inflammation during atherosclerosis. This, together with their production of inflammatory chemokines such as MCP-1, IL-8, and MIP-3*α*, results in further recruitment of monocytes, neutrophils and other inflammatory cells. Macrophage-derived TNF*α* and IL-1*β* also activate the vascular endothelium to upregulate adhesion molecules and chemokines [22, 37] and thus promote monocyte migration as part of a positive feedback loop. Activation of macrophages is also enhanced by IFN*γ* released by NK cells and T cells, and macrophage-derived cytokines, such as IL-12 and IL-15, can in turn drive proatherogenic T cells [38]. In addition to the production of inflammatory mediators, macrophage activation results in the induction of several bactericidal systems such as the NADPH oxidase enzyme. This converts oxygen into the superoxide anion and other free radicals, and these reactive oxygen intermediates (ROIs) are toxic to microbes but can damage host tissue due to their capacity to cause DNA degradation and inactivation of metabolic enzymes, and indeed perpetuate atherosclerosis [39]. Activated macrophages also release nitric oxide (NO) which combined with superoxide, generate peroxynitrite which causes cell injury [39, 40]. Further, myeloperoxidase- (MPO-) generated reactive nitrogen species from monocytes contributes to the conversion of LDL to an atherogenic form [41]. In addition, macrophages express nonspecific esterase, lysosomal hydrolases, and ectoenzymes [42], and secrete an array of cathepsins [43] and matrix metalloproteinases (MMPs) [44] that degrade collagens and the extracellular matrix, likely contributing to thinning of the fibrous cap and plaque instability.

Combined with overzealous macrophage activation in atherosclerotic plaques is the impairment of macrophage functions critical for the control and resolution of inflammation. Indeed, an important function of macrophages under both resting and inflammatory conditions is the rapid uptake of apoptotic cells from tissues, termed efferocytosis. Efferocytosis is mediated by a range of receptors such as CD36 [45] and MerTK [46], and chronic apoptosis of lipid-filled foam cells is combined with defective efferocytosis during atherosclerosis [47], likely contributing to the formation of the necrotic core. Interestingly, the receptors involved in the recognition of apoptotic cells, such as CD36 and *α*v*β*3, may also be involved in the recognition of necrotic cells [48]. This may be pertinent when one considers that the vast majority of cell death in advanced plaques is the result of necrosis, a process that drives inflammation and formation of the necrotic core [49].

Finally, an increasing body of evidence indicates that macrophages have developed several strategies to survive and proliferate in the atherosclerotic plaque, such as the unfolded protein response and AP [50]. In particular, recent investigation into macrophage AP in atherosclerosis has demonstrated another pathway through which these cells contribute to vascular pathology [4–7].

## 4. Autophagy Machinery and Regulation

AP (derived from Greek words, “auto” meaning “self” and “phagy” meaning “to eat”) is an evolutionarily conserved controlled cellular catabolic process involving the delivery of cytoplasmic contents to the lysosomal machinery for ultimate degradation and recycling. In mammalian cells, several types of AP have been identified; they are differentiated on the basis of their physiological functions and the mode of cargo delivery to the lysosomal compartment, such as chaperone-mediated AP, microAP, macroAP, and others [51]. MacroAP has been studied most extensively as compared with other types of AP and this paper will focus on macroAP (herein referred to as “AP”). The AP mechanism involves the formation of characteristic double-membrane vesicles, called autophagosomes or autophagic vacuoles, in which cytoplasmic material is sequestered. The origins of this structure remain incompletely understood; it may be generated from multiple sources including the endoplasmic reticulum (ER) [52, 53], the outer mitochondrial membrane [52, 54], and the plasma membrane [55, 56]. The autophagosomes are targeted to lysosomes to form single-membraned autolysosomes with degradative capacity. During the degradative phase, a series of lysosomal enzymes (e.g., cathepsins and other acid hydrolases) digest the contents of autolysosomes, that are then released to the cytosol for recycling or reuse for anabolic pathways and to get rid of toxic harmful cellular substances [57, 58].

In mammalian systems, basal AP is a continuous process serving as a quality control system to clear and recycle damaged and/or unwanted components of the cell including organelles and protein aggregates. This pathway is stimulated by numerous cellular or subcellular stresses, together with nutrient or growth factor deprivation, reactive oxygen species (ROS), hypoxia, DNA damage, protein aggregates, dysfunctional organelles, or intracellular pathogens to counter the stress for cell survival [58]. AP is mostly considered as a cell survival and cytoprotective process but under chronic stress situations, it is also associated with cell death (hence called “autophagic cell death” rather than “cell death with autophagic features”), though the meaning of AP in these situations remains controversial [59]. It is now well acknowledged that AP can exert a critical and decisive influence on a great variety of human physiological and pathophysiological processes, such as cancer, neurodegenerative disorders and cardiovascular diseases [60]. Moreover, the AP machinery also orchestrates various responses to exogenous stimuli such as microorganisms [61]. For instance, AP plays a key role in the defense against bacterial infection [62, 63]. AP is also required for antigen presentation via major histocompatibility complex (MHC) class II, which plays a key role in immune driven diseases [64], including atherosclerosis [65].

Recently, progress has been made in characterizing the AP protein machinery and signaling cascades. It has been demonstrated that, in mammalian cells, the proteins encoded by AP-related genes (Atg) generally form multiprotein complexes that are crucial for autophagosome formation. The core machinery of mammalian AP incorporates five functional subgroups: (i) the unc-51-like kinase (ULK) complex, including ULK1/2, Atg13, and FIP200, involved in AP induction; (ii) the class III phosphatidylinositol 3-kinase (PI3K) complex, consisting of Vps34, Beclin 1, p150, and Barkor (Atg14-like protein). The lipid kinase activity of Vps34 is indispensable for generating phosphatidylinositol (3)-phosphate (PI3P) at the PAS (phagophore assembly site) for the recruitment of other Atg proteins and the formation of the autophagosome; (iii) two ubiquitin-like protein (Atg12 and LC3) conjugation systems involved in the expansion of autophagosome membranes; (iv) Atg9 and its recycling system that contributes to the delivery of membranes forming the autophagosome; (v) the proteins needed for the fusion between autophagosomes and lysosomes for ultimate degradation. The detailed literature concerning these topics can be found elsewhere [51, 58]. However, several non-Atg proteins and different macromolecular signaling complexes are shown to contribute in the regulation of this process [51, 66, 67].

Mammalian target of rapamycin (mTOR), and particularly its complex 1 (mTORC1), acts as a major checkpoint. In normal conditions (presence of growth factors and nutrients), mTORC1 induces phosphorylation of ULK1/2 and Atg13, which inhibits ULK complex activity and, in turn, AP. Many diverse signals, such as growth factors, amino acids, glucose, energy status, and different forms of stress, regulate the mTOR pathway [68]. In conditions that trigger AP, such as nutrient starvation or stimulation with the antibiotic rapamycin, the mTORC1 serine/threonine kinase activity is inhibited; mTORC1 dissociates from the ULK complex, which becomes active. mTORC1 also incorporates upstream activating signals that inhibit AP via the class I PI3K (and protein kinase B, PKB, also known as Akt) pathway [69]. AMPK (adenosine 5′-monophosphate-activated protein kinase) and Sirtuin1 (Sirt1) also control starvation-induced AP through a coordinated fashion depending upon the energy status (ATP/AMP levels) of the system [70], but at the same time AMPK negatively regulates mTORC1 [71, 72]. A second regulatory step required for the autophagosome formation involves the “Beclin 1 core complex.” There are several Beclin 1 complexes: the UVRAG (ultraviolet irradiation resistant-associated gene) [73] or the Rubicon (RUN domain and cysteine-rich domain containing, Beclin 1-interacting) [74, 75] complex, with UVRAG or Rubicon, in place of Barkor, respectively. These 3 complexes act differently: the Barkor complex has a role in the formation of autophagosomes, the UVRAG complex acts in autophagosome maturation, whereas the Rubicon complex inhibits autophagosome maturation [76, 77]. Moreover, other Beclin 1 binding partners have been shown to modulate AP, including ambra-1 (activating molecule in Beclin 1-regulated AP) [78] or Bif-1 (Bax-interacting factor 1) [79]. Under resting conditions, antiapoptotic Bcl-2 protein family members, such as Bcl-2 and Bcl-X_*L*_), constitutively bind Beclin 1 and act as negative regulators of AP, showing intricate interlinked complex control between AP and apoptosis processes [80–82]. The tumor suppressor gene, *p53*, has been reported to play a dual role in AP [83]. p53 can induce AP through activation of AMPK [84] and upregulation of DRAM (damage-regulated modulator of AP) [85], where cytoplasmic p53 can inhibit AP (transcription independent activity) [86]. The NF-*κ*B transcription factor, and its certain upstream regulators, connects and integrates diverse stress response signals including immune signals with the AP pathway [87–89]. Others regulators that induce AP include tumor suppressors, such as PTEN, TSC1 and TSC2 complexes, and the death-associated kinase (DAPK); stress-activated signaling molecules, such as c-Jun N-terminal kinase 1 (JNK1), and those that respond to endoplasmic reticulum (ER) stress (PERK, eIF2*α*-kinase, and IRE1), and molecules involved in innate immune signaling, such as TLRs and immunity-related GTPases [90].

## 5. AP and Macrophages

In the last decade, it has been established that AP exerts important functions in many aspects of immune and inflammatory responses [91, 92]. AP is under the control of immune receptors and cytokine signaling [91, 92] and is stimulated upon microbial recognition by pattern recognition receptors (PRRs) [93–96] or activation with T helper 1 cytokines [97].

AP plays a critical role in host defense by promoting the elimination of pathogens via autolysosomes (referred to as xenophagy) as well as the delivery of microbial nucleic acids and antigens to endo/lysosomal compartments for activation of innate and adaptive immune responses [98]. *In vitro*, it has been demonstrated that AP has a crucial role in macrophage phagocytosis of different pathogens, such as *Listeria monocytogenes*, *Mycobacterium tuberculosis*, *Herpes simplex virus type I*,* Toxoplasma gondii* and many others [99, 100]. Immunity-related GTPase family M, an IFN-inducible GTPase, promotes AP that is involved in the elimination of mycobacteria in macrophages [101, 102]. Consistent with this, macrophages lacking Atg7 fail to eliminate live yeasts in phagolysosomes [103]. In mice, knockout of Atg5 in macrophages and neutrophils increases susceptibility to infection with *L. monocytogenes* and the protozoan *T. gondii* [104].

It has been shown that AP machinery and pathways interact with several PRRs. Firstly, TLRs were connected with AP [61, 103, 105]. Activated TLRs recruit adaptor proteins such as myeloid differentiation marker 88 (MyD88) and tumor necrosis factor receptor- (TNFR-) associated factor 6 (TRAF6), an E3 ubiquitin ligase and scaffold protein. In macrophages, both MyD88 and another adaptor protein, TRIF (TIR domain-containing adaptor inducing interferon-beta), interact with Beclin 1. Exposure of murine macrophages to a TLR4 ligand (lipopolysaccharide, LPS) also reduces the association between Beclin 1 and Bcl-2. TLR4 triggers AP via TRAF6 that ubiquitinates Beclin 1 and determines the Beclin 1/Bcl-2 dissociation [106]. Furthermore, it has been reported that TLR7-induced AP in murine macrophage cell lines depends on MyD88 and Beclin 1 [96]. The stimulation of TLR2 with zymosan (a cell component of fungi) triggers the recruitment of LC3 to the phagosomes although the signaling pathways seem MyD88-independent [94]. The soluble TLR2 ligand, Pam3CSK4, only when fused with latex beads, is able to induce the maturation of phagosomes in primary macrophages, suggesting that TLR2 signaling is necessary but not sufficient for the induction of AP [94].

Others receptors involved in the detection of invasive pathogens, the cytosolic Nod- (nucleotide-binding oligomerization-domain-) like (NLRs) and RIG-I-like (RLRs) receptors, have been demonstrated to be players in the autophagic response to bacteria [107]. Among the members of the NLR family, Nod1 and Nod2 detect intracellular bacteria through their ability to sense bacterial peptidoglycan [108]. Activation of Nod1 and Nod2 initiates a proinflammatory response dependent mainly on the activation of the transcription factor NF-*κ*B and on the recruitment of the adaptor protein RIP2 [109, 110]. Similar to TLRs, mutations in genes encoding Nod proteins have been associated with chronic inflammatory disorders [111].

Travassos and colleagues demonstrated that, in mouse macrophages and other cell lines, Nod1 and Nod2 recruited the AP protein ATG16L1 to the plasma membrane at the bacterial entry site by a mechanism independent of the adaptor RIP2 and transcription factor NF-*κ*B [107]. In contrast to Nod2, NLRC4 (Ipaf) and NLRP4 exert inhibitory effects on AP. NLRC4 acts negatively at the initiation stage, whereas NLRP4 acts at both the initiation and maturation stages. NLRs, including those essential for inflammasome assembly and activation such as NRLP3, are found in complexes with Beclin 1 in several human cell lines including acute monocytic leukemia cells [112].

Recently, the autophagic adaptors, sequestosome 1/p62-like receptors (SLRSs), have been proposed as a new category of PRRs in order to link AP and innate immunity signaling [113]. The autophagic adaptors, NBR1 and p62, present at the earliest stages of autophagosome formation, have been studied independently of AP as complex inflammatory signaling platforms [114, 115]. A recent study performed with both *Drosophila* blood cells and mouse macrophages show that constitutive p62-mediated selective AP is required for cell spreading and Rho1-induced cell protrusions. It is suggested that p62 may mediate selective autophagic degradation of a regulator of the Rho pathway. Moreover, it is becoming apparent that proteins, organelles, and pathogens can be targeted for autophagic clearance by selective mechanisms, although the extent and roles of such degradation are unclear. These results illuminate a specific and conserved role for AP as a regulatory mechanism for cortical remodeling, with implications for immune cell function [116]. Moreover, AP can drive the rapid cellular changes necessary for proper differentiation. In fact, it has been shown that this process plays a crucial role in monocyte differentiation into macrophages when this differentiation is induced by colony stimulating factor-1 (CSF-1) as well as by M-CSF [117, 118]. SLRs can participate in the promotion of autophagic killing of intracellular microbes. The p62 adaptor protein can deliver specific ribosomal and bulk ubiquitinated cytosolic proteins to autolysosomes where they are proteolytically converted into products capable of killing *M. tuberculosis*. Thus, p62 brings cytosolic proteins to autolysosomes where they are processed from innocuous precursors into neoantimicrobial peptides, explaining in part the unique bactericidal properties of autophagic organelles [119]. However, there are also potential links between AP and conventional antimicrobial peptides, such as cathelicidin, a peptide obtained by conventional proteolysis from larger precursors. Cathelicidin expression and its antimycobacterial action are induced by vitamin D3, an activator of AP, in human monocytes/macrophages [120]. Moreover, vitamin D3 triggers AP in human macrophages that inhibit HIV-1 infection [121].

AP and the inflammasome undergo complex functional interactions. Indeed, damage-associated molecular patterns (DAMPs), toxins, and several particulates and nanomaterials can induce AP directly or via the activation of the inflammasome pathway [113, 122, 123]. Cytokines can induce AP in some cases or, conversely, suppress it in other cases (reviewed in [103, 124, 125]). Some of the immune signals that induce AP include IFN-*γ*, TNF, and CD40-CD40L interactions. In contrast, AP is negatively regulated by the T helper type 2 cytokines, IL-4 and IL-13 [61]. Several observations have revealed a link between autophagic protein deficiency and proinflammatory cytokine secretion in macrophages. Macrophages lacking Atg16L1 and Atg7 produce high amounts of IL-1*β* and IL-18, but not TNF or IFN-*β*, in response to LPS. In these macrophages, the enhanced IL-1*β* production is induced by Toll/IL-1 receptor domain-containing adaptor inducing IFN-*β* (TRIF)-dependent generation of ROS. Further, deletion of Atg5 in macrophages enhances retinoic acid-inducible gene-I-like receptor- (RLR-) mediated type I IFN production in response to single-stranded RNA viruses. These data indicate the importance of Atg in the inflammatory response [103, 126, 127]. Increased activation of IL-1*β* and IL-18 has been observed in macrophages and monocytes genetically deficient in Beclin 1 and LC3B; this occurs through the increased activation of the NLRP3 (NALP3) inflammasome pathway [128, 129]. IFN-*γ* has also been reported to induce AP in macrophage cell lines, but less in primary mouse and human macrophages [97]. Taken together, this evidence suggests that AP in macrophages can be triggered directly via at least some PRRs and indirectly via certain cytokines induced upon PRR activation [130].

In addition, it has been reported that cells undergoing autophagic cell death can induce a proinflammatory response in human macrophages. Indeed, upon engulfing MCF-7 cells undergoing autophagic cell death, human macrophages generate a proinflammatory response involving the secretion of IL-6, TNF*α*, IL-8, and the anti-inflammatory cytokine IL-10 [131]. Interestingly, AP also regulates phagocytosis of dead cells [132, 133]. Importantly, AP can influence adaptive immunity by regulating antigen presentation and the maintenance of lymphocyte function and homeostasis [134].

In conclusion, AP can influence inflammatory responses through several pathways in a cell-intrinsic manner, affecting both pro- and anti-inflammatory signaling and subsequent immune-driven diseases such as atherosclerosis.

## 6. Role of AP in Atherosclerosis

AP in atherosclerosis has been extensively investigated with particular focus on vascular smooth muscle cells (SMCs) and endothelial cells (ECs). Transmission electron microscopy (TEM) of SMCs in the fibrous cap of experimental or human plaques reveals features of AP such as formation of myelin figures [135]. This data is supported by western blot analysis of advanced human plaques showing elevated levels of LC3-II [136].

Several AP triggers are present in the atherosclerotic plaque, such as inflammatory mediators [137], ROS production [138] and accumulation of oxidized LDL [139, 140]. TNF*α* stimulation increases the number of vacuolated cells and the expression of LC3-II and Beclin 1 in SMCs isolated from atherosclerotic plaques [137]. Osteopontin, a protein involved in vascular inflammation, and advanced glycation end products (AGEs) have been shown to induce AP in human and rat SMCs, respectively [141, 142]. Similarly, 7-ketocholesterol, one of the major oxysterols present in atherosclerotic plaques, triggers extensive vacuolization and intense protein ubiquitination and increases the LC3-II expression in cultured SMCs [143]. SMCs in the fibrous cap are surrounded by a thick layer of basal lamina and therefore are subjected to hypoxia, caused by inadequate vascularization [144], and experience nutrient and growth factor deprivation, well-known conditions leading to the induction of AP. Dying SMCs in the fibrous cap of advanced human plaques show ubiquitinated inclusions in their cytoplasm and may undergo autophagic death [143, 145].

AP in vascular endothelial cells can be induced by several compounds in the circulation or in the subendothelial layer of the plaque. For example, oxLDL intensifies AP in human umbilical vein EA.hy926 cells [139], and mitochondrial-derived ROS activate AMPK that in turn increases AP leading to EC survival [146]. AP plays an important role in preserving vascular endothelial function by reducing oxidative stress, increasing nitric oxide bioavailability and reducing vascular inflammation [147]. Importantly, AP is reduced with ageing in vascular tissues [147].

The general consensus is that basal AP can protect plaque cells against oxidative stress by degrading damaged intracellular material [148] and promoting cell survival. The protective role of AP in stabilizing the plaque was confirmed *in vitro* showing that SMC death induced by low concentrations of statins was attenuated by the AP inducer 7-ketocholesterol [149]. Similarly the exposure of ECs in culture to oxLDL or AGEs [139, 150] induced AP which protected against EC injury [151]. Moreover, Salabei et al. [152] showed that the antiproliferative effect of verapamil in SMCs, beneficial in controlling vascular-injury-induced neointimal formation, was associated with the onset of AP. Although verapamil strongly upregulated AP, it did not promote SMC cell death yet appeared to suspend cell division resulting in an anti-proliferative state.

In contrast to basal AP, excessive stimulation of AP in SMCs or ECs may cause autophagic cell death [151], leading to reduced synthesis of collagen, thinning of the fibrous cap, plaque destabilization, lesional thrombosis, and acute clinical events [136].

## 7. Macrophage AP in Atherosclerosis

The investigation of macrophage AP in atherosclerosis has been complicated by the strong phagocytic activity of these cells. It is difficult to determine via conventional electron microscopy whether the vacuoles in their cytoplasm result from autophagocytosis or heterophagocytosis [136, 151]. In addition, the autophagosomal marker protein LC3 is poorly expressed in macrophages and overexpression of other lysosomal marker proteins may give rise to false-positive signals in immunoelectron microscopy [136, 151].

Importantly, pharmacological modulation of macrophage AP has been shown to affect vascular inflammation. Stent-based delivery of everolimus (mTOR inhibitor; a well-known AP inducer; see Section 8) in atherosclerotic plaques of cholesterol-fed rabbits leads to a marked reduction of macrophages via autophagic cell death without altering the SMC plaque content [4]. The observed macrophage cell death was characterized by bulk degradation of long-lived proteins, processing of LC3, and cytoplasmic vacuolization, which are all markers of AP [4]. Further, local administration of imiquimod (TLR7 ligand) to rabbit atherosclerotic carotid arteries induced macrophage AP, without affecting SMCs and ECs. However, in this case the induction of macrophage AP triggered cytokine production and the upregulation of VCAM-1 and enhanced leukocyte infiltration in the artery [5]. The authors speculate that the moderate AP induced in the macrophages of imiquimod-treated lesions does not lead to cell death but to plaque inflammation. Of note, TLR7 stimulation could lead to the activation of other immune cells homing to atherosclerotic vessels such as plasmacytoid dendritic cells, recently shown to play a key role in promoting experimental atherosclerosis in mice [153–155].

Two recent elegant papers add new dimensions to the understanding of the role of macrophage AP in regulating atherosclerotic plaque development [6, 7]. Razani et al. [6] demonstrated that during lesion formation, autophagic markers (p62 and LC3/Atg8) were expressed in the atherosclerotic plaques of apolipoprotein-E (apoE)^−/−^ mice, colocalizing mainly with monocyte-macrophages (MOMA-2, CD11b +ve) and plaque leukocytes (CD45 +ve). The AP protein, p62/SQSTM1, is known to accumulate when autophagy flux through lysosomes is defective [156]. AP induction by prolonged fasting resulted in decreased levels of p62 protein in the aorta of apoE^−/−^ mice, suggesting that changes in aortic p62 protein reflect *in vivo* AP status. p62 levels are raised with increasing age/plaque burden in atherosclerotic aortas, suggesting that initially AP is functional and becomes severely compromised with disease progression.

Beclin 1/Atg6 heterozygous-deficient (Beclin-Het) mice on the apoE^−/−^ background showed similar extent of atherosclerosis compared to apoE^−/−^ mice that were wild-type at the Beclin locus, demonstrating that autophagy haploinsufficiency had no effect on pathology. On the contrary, complete deficiency of macrophage AP increased vascular inflammation and plaque formation. To pursue this notion, the authors used macrophage-specific Atg5-null (Atg5-m*ϕ*KO) mice with complete absence of an AP gene in macrophages. Plaque formation, serum IL-1*β* levels, and aortic IL-1*β* expression were all increased in Atg5-m*ϕ*KO/apoE^−/−^ mice fed a high-fat diet (HFD) as compared to apoE^−/−^ controls. In addition, AP deficiency was associated with elevated plaque macrophage content.

Given the increased levels of IL-1*β* observed in Atg5-null mice, the authors suggest a link between AP deficiency and inflammosome hyperactivation. Indeed, deficient AP, through mechanisms that might include lysosomal leakage, generation of ROS, and impaired mitophagy, could result in the activation of the inflammasome. However, the effects of AP on IL-1*β* production are complex and context dependent and the assumption that AP suppresses the inflammosome in vascular inflammation merits further investigation.

The protective role of macrophage AP in atherosclerosis was confirmed by Liao et al. [7]. The authors provide evidence that AP prevents macrophage apoptosis and defective efferocytosis, both promoting plaque necrosis in advanced atherosclerosis. Firstly, authors demonstrated, in primary macrophages from mice transgenic for a GFP-tagged version of the AP effector LC3-II, that several proatherosclerotic stimuli induced AP and promoted autophagic flux through lysosomes. Inhibition of AP by silencing Atg5 or in Atg5-deficient macrophages enhanced apoptosis and NADPH oxidase-mediated oxidative stress, rendering the apoptotic cells less recognizable to efferocytosis. Importantly, the same findings were confirmed *in vivo*. Aortic root lesions of HFD-fed GFP-LC3/low-density lipoprotein receptor (LDLr)^−/−^ mice contained macrophages expressing Atg5 and displaying the punctate pattern of GFP-LC3 fluorescence typical of AP. The number of macrophages expressing p62 increased as lesions progressed. These data confirm the presence of AP in atherosclerotic vessels and suggest that autophagic flux through lysosomes decreases as disease progresses. Lesion size and necrotic area were higher in Atg5^fl/fl^Lysmcre^+/−^/LDLr^−/−^ versus control mice. The number of lesional macrophages was not increased, however, macrophage-rich regions of the Atg5^fl/fl^Lysmcre^+/−^/LDLr^−/−^ plaques had more apoptotic cells positive for TUNEL, activated caspase-3, DHE, and p47. In summary, macrophage AP deficiency increased apoptosis and oxidative stress in plaque macrophages, promoted plaque necrosis, and impaired lesional efferocytosis in LDLr^−/−^ mice. These data complement results obtained by Razani et al. [6], confirming a protective role played by macrophage AP in the two most widely used mouse models of atherosclerosis.

Another important aspect to consider is the contribution of lipophagy to vascular pathology. As recently reviewed [157–159], lipophagy, a special kind of AP, contributes in cholesterol egress from lipid-laden cells to high-density lipoprotein (HDL) via lysosomal lipases. AP can play a role in the hydrolysis of stored cholesterol droplets in macrophages, thus facilitating cholesterol efflux [160]. Interestingly, Wip1 phosphatase, a known negative regulator of Atm-dependent signaling, has been recently shown to play a major role in controlling AP and cholesterol efflux in apoE^−/−^ mice. Deletion of Wip1 resulted in suppression of macrophage conversion into foam cells, thus preventing the formation of atherosclerotic plaques [161].

In conclusion, macrophage AP becomes dysfunctional in atherosclerosis and its deficiency promotes vascular inflammation, oxidative stress, and plaque necrosis, suggesting a mechanism-based strategy to therapeutically suppress atherosclerosis progression.

## 8. Pharmacological Manipulation of Autophagic Pathways

Several pharmacological agents that are able to modulate AP have already been identified, such as mTOR inhibitors, AMPK modulators, IP_3_ and calcium lowering agents, and lysosome inhibitors [162, 163].

mTOR inhibitors are the most studied AP inducers, and among them, rapamycin (also known as sirolimus) was the first drug to be identified. Rapamycin, a lipophilic macrolide antibiotic already in use to prevent the rejection of transplanted organs and to block restenosis after angioplasty, binds to the immunophilin FK506-binding protein of 12 kDa (FKBP12) inhibiting the kinase activity of mTOR, particularly TORC1 [164, 165]. AP induction with rapamycin enhances the clearance of toxic substrates, such as intracellular aggregate-prone proteins associated with neurodegenerative diseases, and protects against toxicity of these substrates in cell and animal models [166–168]. However, rapamycin also regulates numerous physiological processes that are independent of AP [68]. Indeed, rapamycin inhibits the translation of numerous proteins, causes immunosuppression and cell cycle arrest and alters cell size [169]. These side effects may be unwelcome consequences if rapamycin is used as an AP enhancer, and consequently there is a need for nonimmunosuppressive AP-inducing drugs. Of note, rapamycin and its analogs (temsirolimus, everolimus, and deforolimus) have had limited success as anticancer drugs, may be because they inhibit mTORC1, but not mTORC2 [170]. Consequently, a more complete blockade of the mTOR pathway has led to the development of ATP-competitive mTOR inhibitors of both mTORC1 and mTORC2 (e.g., PP242, AZD8055, WYE132, and Torin 1). Although these compounds clearly show preclinical evidence of antitumor activity, their effectiveness in the clinical setting has yet to be demonstrated [171–174].

Recently, further mTORC1 inhibitors, such as perhexiline, niclosamide, and rottlerin, have been identified as compounds that increase autophagosome number but further work is required to completely understand their activity [175].

Interestingly, several other drugs with well-known pharmacological actions can induce AP by mTOR-independent pathways. For example, mood-stabilizing drugs, such as carbamazepine, valproic acid, and lithium have been identified as AP inducers by reducing IP_3_ levels [176, 177]. Furthermore, L-type calcium channel antagonists (e.g., verapamil), and antiarrhythmic drugs (e.g., amiodarone) induce AP by inhibiting levels of calcium [162]. The anticancer drug tamoxifen appears to function in part by upregulating the level of Beclin 1 and inducing AP [178]. Finally, the antidiabetic drug metformin has been shown to induce AP of several cancer cell types by activating AMPK [179–181].

A useful strategy for pharmacological manipulation of AP based on additive effects of drugs could be obtained using mTOR inhibitors in combination with mTOR-independent AP enhancers. It has been demonstrated that trehalose and small molecule enhancer rapamycin (SMERs) exerted an additive effect on the clearance of aggregate-prone proteins associated with Huntington's disease and Parkinson's disease when associated to rapamycin [168, 182].

AP inhibitors can be classified in: (i) early stage inhibitors including 3-methyadenine (3-MA), wortmannin and LY294002 that target the class III PI3K; (ii) late stage inhibitors, including chloroquine (CQ) or hydroxychloroquine (HCQ), bafilomycin A1, and monensin that prevent fusion of autophagosomes with the lysosomes [183]. Currently, several ongoing clinical trials registered with the National Cancer Institute (http://www.cancer.gov/clinicaltrials) are evaluating the efficacy of the combination of HCQ with cytotoxic drugs in a variety of cancers.

Importantly, many of the aforementioned pharmacological agents have been shown to be effective in the treatment of cardiovascular disorders, including cardiomyopathy, and heart failure in which AP is involved [184]. For example, reduction of infarct size has been demonstrated in mice treated with rapamycin [185]. Rapamycin, AICAR, and metformin improve cardiac function, reduce cardiac hypertrophy, and delay the onset of heart failure during overload pressure [186, 187]. On the other hand, a strong activation of AP, due to Beclin 1 upregulation, is observed in response to severe pressure overload, and this could be responsible for the transition from compensatory ventricular hypertrophy to pathological remodeling [188]. Finally, several indications support AP as a therapeutic target in experimental atherosclerosis [189]. Sirolimus and everolimus are antiatherogenic in mice [190–193]; on the other hand, AP induction by calorie-deprivation reduced atherosclerosis [194]. However, no clinical data are available regarding the efficacy of AP modulators in cardiovascular diseases.

In conclusion, several drugs that regulate AP have been identified, suggesting that the autophagic signaling may be manipulated to treat human disease. However, considering the dual role of AP in cytoprotection and cell death, there is a need for more specific molecules in order to target the pathways that control AP.

## 9. Conclusions

This paper summarizes recent evidence showing a protective role played by macrophage AP in atherosclerosis. AP becomes dysfunctional in atherosclerosis and its deficiency promotes vascular inflammation, oxidative stress, and plaque necrosis. However, further work is needed to obtain a better understanding of this phenomenon in all stages of the pathology. We need to understand how AP is induced in atherosclerotic lesions, how lipophagy contributes to the pathology, the mechanisms governing the crosstalk between AP and apoptosis within the arterial wall, how they could influence plaque stability, and if they may prove to be effective therapeutic targets.

What degree of AP deficiency is proatherogenic? Is AP induction anti-atherogenic? The answer to these questions requires further investigation. Importantly, to date no studies have addressed the potential effect of AP on the multiple leukocyte subsets which have been shown to infiltrate the naïve and inflamed vessels playing a significant role in plaque formation and development [1, 195–197]. For example, AP regulates antigen presentation [198], T-cell development and homeostasis [113, 199], T-cell and dendritic cell activation [200, 201], and degranulation of mast cells [202]. However, none of these AP functions have been investigated in the context of vascular inflammation. As these studies progress we can expect to learn more about whether AP is indeed a good target for therapeutic intervention in atherosclerosis.
